# Chronic and acute effects of walnuts on antioxidant capacity and nutritional status in humans: a randomized, cross-over pilot study

**DOI:** 10.1186/1475-2891-9-21

**Published:** 2010-05-12

**Authors:** Diane L McKay, C-Y Oliver Chen, Kyung-Jin Yeum, Nirupa R Matthan, Alice H Lichtenstein, Jeffrey B Blumberg

**Affiliations:** 1 Jean Mayer US Department of Agriculture Human Nutrition Research Center on Aging at Tufts University, 711 Washington St, Boston, MA 02111, USA

## Abstract

**Background:**

Compared with other common plant foods, walnuts (*Juglans regia*) are consistently ranked among the highest in antioxidant capacity. In vitro, walnut polyphenols inhibit plasma and LDL oxidation, while in animal models they lower biomarkers of oxidative stress and raise antioxidant capacity. A limited number of human feeding trials indicate that walnuts improve some measures of antioxidant status, but not others.

**Methods:**

A 19 wk, randomized crossover trial was conducted in 21 generally healthy men and postmenopausal women ≥50 y to study the dose-response effects of walnut intake on biomarkers of antioxidant activity, oxidative stress, and nutrient status. Subjects were randomized to receive either 21 or 42 g raw walnuts/d during each 6 wk intervention phase with a 6 wk washout between phases. Subjects were instructed to consume their usual diet, but refrain from eating any other tree nuts, seeds, peanuts, or ellagitannin-rich foods during the entire study, and other polyphenol-rich foods for 2 d prior to each study visit.

**Results:**

Compared to baseline levels, red blood cell (RBC) linoleic acid and plasma pyridoxal phosphate (PLP) were significantly higher after 6 wk with 42 g/d walnuts (P < 0.05 for both). Overall, changes in plasma total thiols, and other antioxidant biomarkers, were not significant with either walnut dose. However, when compared to fasting levels, plasma total thiols were elevated within 1 h of walnut consumption with both doses during the baseline and end visits for each intervention phase (P < 0.05 for all). Despite the observed increase in RBC linoleic and linolenic acids associated with walnut consumption, this substrate for lipid peroxidation only minimally affected malondialdehyde (MDA) and antioxidant capacity. The proportional changes in MDA and Oxygen Radical Absorbance Capacity (ORAC) were consistent with a dose-response effect, although no significant within- or between-group differences were observed for these measures.

**Conclusions:**

Walnut consumption did not significantly change the plasma antioxidant capacity of healthy, well-nourished older adults in this pilot study. However, improvements in linoleic acid and pyridoxal phosphate were observed with chronic consumption, while total plasma thiols were enhanced acutely. Future studies investigating the antioxidant effects of walnuts in humans are warranted, but should include either a larger sample size or a controlled feeding intervention.

**Trial Registration:**

ClinicalTrials.gov: NCT00626691

## Background

Epidemiological studies consistently show that increased consumption of plant-based, antioxidant-rich foods, i.e., fruits, vegetables, whole grains, and nuts, is associated with the reduced risk for several chronic diseases. Clinical trials have demonstrated that a nut-containing diet low in saturated fat and cholesterol, while high in poly- and monounsaturated fatty acids, has a beneficial effect on plasma lipids and lipoproteins when compared with either a low fat or average American diet [1]. However, the LDL cholesterol-lowering response shown in these trials is greater than expected based on equations derived from dietary fatty acid profiles, and may not be solely due to the fatty acid composition of nuts [2]. Other bioactive compounds present in nuts, including micronutrients, fiber, and phytochemicals, may also contribute to their cardio-protective effect by reducing inflammation, improving vascular reactivity, and lowering oxidative stress [2].

Among common plant foods consumed worldwide, walnuts (*Juglans regia*) were ranked second only to rose hips (*Rosa canina*) in their antioxidant activity, as determined by the ferric reducing antioxidant power (FRAP) assay [3]. Compared with other tree nuts, walnuts were also ranked highest when measured with the FRAP, total radical-trapping antioxidant parameter (TRAP), and Trolox equivalent antioxidant capacity (TEAC) assays [4]. Most of this antioxidant activity can be attributed to the polyphenolic constituents, including the ellagitannins, present primarily in the pellicle [5].

Polyphenols isolated from walnuts, including ellagic acid monomers, polymeric tannins, and other phenolic compounds, are potent inhibitors of plasma and LDL oxidation in vitro [6], and have been found to decrease biomarkers of oxidative stress in diabetic mice [7]. Melatonin, another antioxidant constituent present in walnuts, has been positively correlated with increased plasma antioxidant capacity in rats [8]. A limited number of human feeding trials, conducted in subjects at high risk for CVD, indicate that walnuts improve endothelial function [9], and affect some measures of antioxidant status [10], but not others [11].

The goal of this pilot study was to determine whether consuming walnuts in amounts readily incorporated into the diet can affect the plasma antioxidant capacity and nutrient status of healthy older adults in a dose-dependent manner. There are many different kinds of antioxidants differentially distributed in blood, such that each assay currently available quantifies the overall contribution of each antioxidant in a different way [12]. Thus, employing a panel of established assays should more accurately quantify the overall antioxidant response to a walnut intervention.

## Methods

### Study Design and Intervention

A 19 wk, randomized crossover trial was conducted in 21 generally healthy men and postmenopausal women ≥50 y to study the dose-response effects of chronic walnut intake on biomarkers of antioxidant activity, oxidative stress, and nutrient status. Subjects were asked to refrain from eating any other tree nuts, seeds, peanuts, or ellagitannin-rich foods (including berries, wine, cocoa, tea, and pomegranates) during the entire 19 wk study, and other polyphenol-rich foods (including most fruits, vegetables, beans, legumes, whole grains, olive oil and coffee) for 2 d prior to each study visit. The trial consisted of a 1 wk run-in period, two 6 wk intervention phases, and a 6 wk washout period between interventions. Subjects were randomized to receive either 21 or 42 g packaged raw walnuts/d during the first intervention phase and the alternate dose during the second intervention phase. The doses were based on the FDA qualified health claim that the regular consumption of 1.50 oz (42 g)/d walnuts decreases the risk of heart disease. Packaged walnuts were provided by the California Walnut Commission (Folsom, CA). Fasting plasma samples were collected at the baseline and end of each 6 wk phase. Plasma samples were also collected 1 h post-walnut consumption (included in a standard meal) at each of these visits. Randomization was stratified by gender according to a computer generated list. Study personnel were blinded to the treatment assignment for the duration of the intervention and sample analysis. The only exception was the study dietitian who was responsible for distributing the walnuts and dietary instructions to eligible subjects at randomization, and assessing compliance. During each visit, subjects reported to the Jean Mayer USDA Human Nutrition Research Center on Aging (HNRCA) at Tufts University after fasting for 12 h. At each visit, subjects were queried regarding interval changes in health, as well as use of prescription medications, tobacco, and dietary supplements.

### Subject eligibility

Twenty-one non-smoking men and postmenopausal women, age ≥50 y, with body mass index (BMI) 18.5-35 kg/m^2 ^were recruited from the greater Boston area via newspaper advertisements, direct mailings, and clinic postings. Subjects were excluded if they had a history or known allergy to nuts of any kind; regularly consumed ≥5 oz (140 g) nuts/wk within 6 wk of study admission; were taking estrogen, oral steroids or cholesterol-lowering medications; had renal, endocrine or gastrointestinal disease, rheumatoid arthritis; presented with systolic blood pressure >150 mm Hg and/or diastolic blood pressure >95 mm Hg; had usual ethanol intake ≥2 drinks (28 g)/d or used illicit drugs; presented with EKG or standard clinical laboratory values outside acceptable parameters. Subjects were also excluded if they used dietary supplements containing either fish oils, high doses (≥3× U.S. Daily Value) of vitamins C, E, β-carotene or selenium, and/or phenolic compounds (e.g., herbal or berry-containing preparations) within 6 wk of study admission.

In total, 23 volunteers were recruited for an initial baseline screening visit between February and October 2007. Two subjects declined to participate after the dietary restrictions were reviewed, but before the start of the first 6 wk period. No subject dropped out of the study prior to completion. The study design was approved by the Institutional Review Board of Tufts University Health Sciences Campus and Tufts Medical Center. All subjects signed a written informed consent agreement before participating.

### Sample Collection and Preparation

Collected samples were assessed for selected measures of antioxidant activity (ORAC, ORAC with perchloric acid (pca) precipitation, FRAP, and Total Antioxidant Performance [TAP]), lipid peroxidation (MDA), biomarkers of antioxidant status (total thiols, phenols, carotenoids, and glutathione peroxidase [GPX]), micronutrients (α- and γ-tocopherol, folate, pyridoxal phosphate, vitamin C, magnesium), RBC fatty acid profiles, and lipid profile (total, low density lipoprotein [LDL] and high density lipoprotein [HDL] cholesterol, and triglycerides) in fasting plasma at the baseline and end of each walnut intervention phase. Data were also collected on ORAC, ORAC-pca, FRAP, total phenols, and thiols in plasma 1-h after walnut consumption at each visit during the intervention.

Blood samples for the analysis of FRAP, TAP, MDA, total thiols, phenols, fatty acids, lipids, folate, PLP, carotenoids, and vitamins C and E were collected in EDTA-containing evacuated tubes and centrifuged within 15 min of drawing (3000 × g, 15 min, 4°C) with a SUR-Sep cap (Organon Teknika, Durham, NC). Samples for the ORAC and plasma glutathione peroxidase assays were collected in heparin tubes, while samples for the analysis of magnesium were collected in trace metal-free tubes (Becton, Dickinson, and Company, Franklin Lakes, NJ) and processed similarly. Samples for MDA analysis were prepared by adding 10 μL of 50 μM butylated hydroxytoluene (BHT) to 250 μL of plasma. An equal volume of cold 0.35 M perchloric acid was mixed with the plasma sample for vitamin C analysis, followed by centrifugation (2500 × g, 10 min, 4°C). For the fatty acid analyses, packed red blood cells (RBC) were washed with saline and BHT added at 0.4 mg/mL to prevent oxidation. Sample aliquots were stored in 2 mL NUNC tubes (Vanguard Cryotubes, Neptune, NJ) at -80°C. Aliquots for the magnesium analysis were stored in acid-washed storage tubes also at -80°C. All samples for each subject were analyzed within the same run for every assay performed.

### Biochemical Analyses

#### Antioxidant activity markers

The ORAC values of whole plasma and perchloric acid treated protein-free plasma were determined according to the method of Huang et al. [13] and Cao et al. [14]. The assay was carried out on a FLUOstar OPTIMA plate reader using a fluorescence filter with an excitation wavelength of 485 nm and an emission wavelength of 520 nm with the data expressed as μmol Trolox Equivalents (TE)/L. The FRAP value of whole plasma was determined with the spectrophotometric method of Benzie [15] with the data expressed as μmol TE/L. Serum TAP was determined by the method developed by Aldini et al. [16] to measure total antioxidant capacity in both the hydrophilic and lipophilic compartments of serum, and validated by Beretta et al. [17] for the application to high throughput studies. This method measures the rate of oxidation of 4,4-difluoro-5-(4-phenyl-1,3-butadienyl)-4-bora-3a,4a-diaza-s-indacene-3-undecanoic acid (BODIPY 581/591), a lipid-soluble fluorescent probe, and uses the lipid-soluble radical initiator 2,2'-azobis(4-methoxy-2,4-dimethylvaleronitrile) (MeO-AMVN). Oxidation is determined by monitoring the appearance of green fluorescence of the oxidation product of BODIPY with an excitation wavelength of 500 nm and emission wavelength of 520 nm using a 1420 multilabel counter (Wallac Victor 2, Perkin Elmer Life Sciences, MA). The results are expressed as TAP values, which represent the percentage of inhibition of BODIPY oxidation in human serum with respect to that occurring in a control sample consisting of BODIPY 581/591 in phosphatidylcholine liposomes.

#### Lipid peroxidation

Plasma MDA was measured by HPLC with fluorometric detection [18] with the data expressed as μmol/L.

#### Antioxidant biomarkers

Total thiols in plasma were determined spectrophotometrically according to Hu [19] and the data expressed as mmol/L. The total phenol content of plasma was determined by the Folin-Ciocalteu reaction according to Singleton et al. [20] with the data expressed as μmol gallic acid equivalents (GAE)/L. Total carotenoids were determined spectrophotometrically according to the method described by Roels et al. [21]. GPX activity was assessed using a kinetic assay with UV detection according to Pleban et al. [22].

#### Plasma micronutrients

A reverse-phase gradient HPLC method was used for the simultaneous determination of α- and γ-tocopherol [23]. PLP was measured with a radioenzymatic assay described by Camp et at. [24]. Folic acid was assessed with a chemiluminescent immunoassay (Diagnostic Products Corporation, Los Angeles, CA). Vitamin C was measured with an isocratic reverse-phase HPLC procedure according to Behrens et al. [25]. Magnesium was determined with a clinical chemistry analyzer (Olympus AU400, Center Valley, PA) according to the manufacturers' instructions.

#### Fatty acids

RBC fatty acid profiles were quantified using an established gas chromatography method as previously described [26,27]. Peaks of interest were identified by comparison with authentic fatty acid standards (Nu-Check-Prep, Elysian, MN) and the data expressed as molar percentage (mol %) proportions of fatty acids relative to the internal standard.

#### Lipids

Plasma concentrations of total cholesterol, LDL, HDL, and triglycerides were determined with a clinical chemistry analyzer (Olympus AU400, Center Valley, PA) according to the manufacturers' instructions.

### Dietary Assessment

Dietary assessments were made at the baseline and end of each 6 wk intervention phase using a validated food frequency questionnaire (Fred Hutchinson Cancer Research Center Food Frequency Questionnaire Version 06.10.88, Cancer Prevention Research Program, Fred Hutchinson Cancer Research Center, Seattle, WA) to determine usual nutrient intakes and detect any significant changes that may have occurred during the intervention periods.

### Compliance

Compliance was measured by having each subject keep track of their walnut intake using a daily diary chart, and by having the study dietitian count opened bags at each visit. Compliance with the walnut regimen was determined to be >90%.

### Statistical analyses

Statistical analyses were performed using SPSS version 15.0 (SPSS, Inc., Chicago, IL). All outcome variables were tested for sequence and dose effects using a linear mixed model; none were found. Paired t-tests were used to compare changes from baseline values for each respective walnut dose at 1 h post-consumption and after 6 wk of daily consumption, and to compare changes between the two walnut doses achieved after each 6 wk intervention. Regression was used to assess the independent predictive capability of the plasma micronutrients on antioxidant activity measures. Student's t test and chi-square analyses were used to compare baseline characteristics between the two groups at randomization. Unless otherwise noted, results are expressed as either mean ± SD or % change from baseline with respective P values for within and between group differences. P values < 0.05 were considered statistically significant.

## Results

The nutrient content of each walnut dose is shown in Table 1[28]. Subjects' clinical characteristics and nutrient status at randomization are presented in Table 2. No significant changes in body weight were observed during either intervention. Baseline dietary intake data is presented in Table 3. Data from subsequent food frequency questionnaires revealed no significant differences in subjects' dietary intake within or between each intervention phase.

**Table 1:** Major nutrients present in walnuts^1^

	Quantity per dose	
	
Nutrient	21 g	42 g
Calories (kcal)	139	278
Total fat (g)	14	28
Fatty acids, 18:2 (g)	8.6	17.1
Fatty acids, 18:3 (g)	2	4.1
α-Tocopherol (mg)	0.2	0.3
γ-Tocopherol (mg)	4.4	8.9
Vitamin B6 (mg)	0.1	0.2
Folate (μg)	21	42
Magnesium (mg)	34	67

^1^Adapted from [28]

**Table 2:** Subject characteristics at baseline^1,2^

	Dose at randomization	
	
	21 g	42 g
Age, y	64.8 ± 7.8	56.6 ± 13.9
BMI, kg/m2	27.3 ± 3.6	27.4 ± 3.8
Female, % (n)	55 (6)	60 (6)
Male, % (n)	45 (5)	40 (4)
Systolic BP, mm Hg	109 ± 7	116 ± 14
Diastolic BP, mm Hg	73 ± 10	72 ± 10
TC, mmol/L	5.49 ± 1.17	5.54 ± 1.06
LDL, mmol/L	3.37 ± 0.96	3.50 ± 0.91
HDL, mmol/L	1.61 ± 0.44	1.48 ± 0.18
Triglycerides, mmol/L	1.21 ± 0.40	1.24 ± 0.53
Total carotenoids, μmol/L	2.2 ± 1.0	2.6 ± 1.2
α-tocopherol, μmol/L	26.4 ± 6.8	29.3 ± 7.1
γ-tocopherol, μmol/L	5.4 ± 3.1	4.4 ± 2.7
Vitamin C, μmol/L	48.3 ± 21.0	60.8 ± 30.1
Folate, nmol/L	56.0 ± 17.2	50.1 ± 17.4
PLP, nmol/L	44.6 ± 17.7	67.8 ± 59.7

^1^No significant between-group differences were detected using the independent samples t-test (continuous variables) or chi-square test (categorical variables).

^2^Values are means ± SD or n (%)

**Table 3:** Subjects' baseline daily dietary intake^1,2^

	Dose at randomization	
	
Nutrient	21 g	42 g
Energy (kcal)	1638 ± 440	1466 ± 458
Protein (g)	77 ± 35	77 ± 33
Carbohydrate (g)	177 ± 57	168 ± 41
Fat (g)	68 ± 26	54 ± 26
Cholesterol (mg)	294 ± 209	328 ± 262
Fiber (g)	13 ± 5	14 ± 6
Thiamin (mg)	1.3 ± 0.4	1.2 ± 0.4
Riboflavin (mg)	2.1 ± 1.2	2.0 ± 0.6
Niacin (mg)	18 ± 5	18 ± 6
Folate (μg)	254 ± 100	308 ± 116
Vitamin B6 (mg)	1.7 ± 0.6	1.7 ± 0.4
Vitamin B12 (mg)	6.1 ± 3.1	7.3 ± 4.5
Vitamin C (mg)	92 ± 56	115 ± 43
Retinol (μg)	538 ± 356	574 ± 381
β-carotene (μg)	2056 ± 1175	3232 ± 1712
Total tocopherols (mg)	7.0 ± 1.9	7.8 ± 3.6
α-tocopherol (mg)	5.7 ± 1.8	6.6 ± 3.4
γ-tocopherol (mg)	12.7 ± 6.1	10.9 ± 8.0
Vitamin D (μg)	7.8 ± 5.8	6.3 ± 2.9
Calcium (mg)	1011 ± 658	964 ± 415
Magnesium (mg)	284 ± 98	281 ± 77
Potassium (mg)	2826 ± 1028	2970 ± 805
Sodium (mg)	2697 ± 910	2705 ± 1068
Iron (mg)	12 ± 4	13 ± 5
Zinc (mg)	12 ± 5	12 ± 5
Selenium (μg)	100 ± 36	102 ± 58

^1^No significant between-group differences were detected using the independent samples t-test (continuous variables).

^2^Values are means ± SD

The main study outcomes are presented in Table 4. After 6 wk, the magnitude of change in plasma ORAC, ORAC-pca, and FRAP levels was higher with 42 g/d walnuts than with 21 g/d, while the degree of lipid peroxidation, assessed by MDA production, was lower. Although no significant within- or between-group changes were observed for any of these antioxidant activity measures (including TAP), the proportional changes in ORAC and MDA with each walnut dose were consistent with a dose-response effect. Compared to the number of subjects who responded to the low dose, a higher proportion of subjects responded with an increase in plasma ORAC, ORAC-pca, and FRAP following the higher walnut dose. The percent of subjects with increased antioxidant activity after 6 wk with 42 vs. 21 g/d was 71 vs. 48% for ORAC, 67 vs. 43% for ORAC-pca, and 38 vs. 19% for FRAP, respectively.

**Table 4:** Changes in antioxidant activity following 6 wk intervention with daily walnut consumption^1^

	Walnut intervention dose				
		
	21 g/d		42 g/d		
			
Measure	% Change from baseline	P (within group)	% Change from baseline	P (within group)	P (between group)
Antioxidant capacity:					
ORAC	2.3 ± 16.4	0.534	5.6 ± 15.5	0.115	0.435
ORAC-pca	-2.1 ± 29.6	0.752	8.2 ± 29.4	0.218	0.322
FRAP	-3.1 ± 10.6	0.197	-0.8 ± 12.1	0.768	0.503
TAP	0.9 ± 6.8	0.572	-0.7 ± 9.7	0.745	0.505
Lipid peroxidation biomarker:					
MDA	8.2 ± 40.6	0.367	2.1 ± 27.9	0.734	0.522
Antioxidant status:					
Total phenols	-2.0 ± 7.0	0.202	-2.0 ± 5.9	0.135	0.995
Total thiols	-2.2 ± 13.2	0.476	1.8 ± 32.2	0.813	0.680
GPX	-1.3 ± 7.8	0.452	-2.4 ± 7.5	0.164	0.646

^1^Values are means ± SD

**Figure 1:**
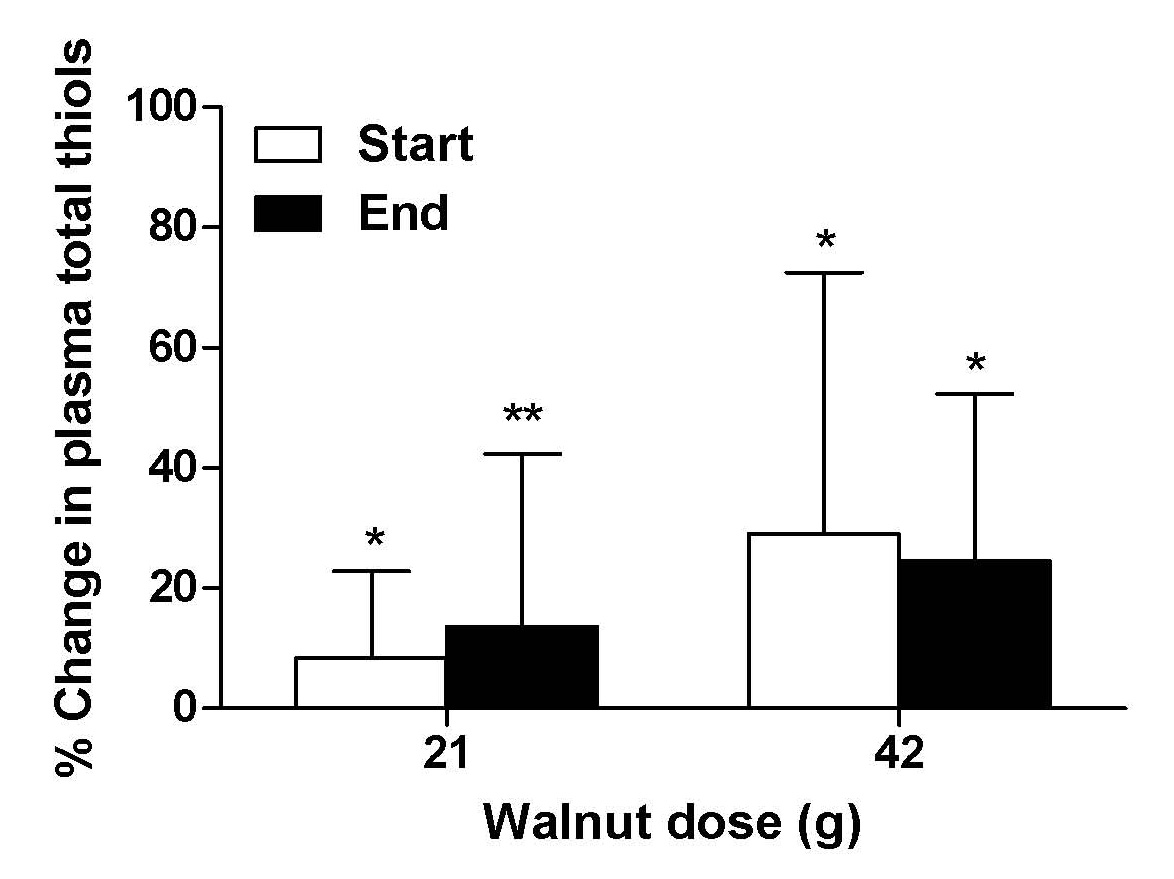
**Acute changes in plasma total thiols within 1 h of walnut consumption**. Mean total plasma thiols just prior to consuming a single 21 g dose were 2.90 ± 0.52 and 2.81 ± 0.41 mM, respectively, during the start and end of intervention visits for this phase. Mean total plasma thiols just prior to a single 42 g dose were 2.65 ± 0.63 and 2.55 ± 0.38 mM during the start and end visits for this phase. Changes in plasma total thiols were measured 1 h following walnut consumption, and compared with the respective baseline values at each visit using paired t-tests. P > 0.05 for between group differences, * P < 0.05 for change from baseline values, **P = 0.058 for change from baseline.

Following 6 wk of daily walnut consumption, the changes in plasma total thiols and other antioxidant biomarkers, including total phenols and GPX, were not significant for either dose (Table 4). However, compared to fasting levels, plasma total thiols were elevated within 1 h of walnut consumption with both walnut doses during the baseline and end visits for each intervention phase (Fig 1). No improvements in other measures assessed within 1 h of consumption (total phenols, ORAC, ORAC-pca or FRAP) were observed (data not shown), except for a 3.1% increase in total phenols within 1 h of walnut consumption during the baseline visit for the 42 g dose (P = 0.063 compared with fasting plasma levels).

Changes in plasma micronutrient status are presented in Table 5. No significant within- or between-group changes were observed with respect to the antioxidant nutrients, i.e., total carotenoids, tocopherols, and vitamin C. Adjusting the changes in antioxidant activity measures, i.e., ORAC, ORAC-pca, FRAP, TAP, and MDA for changes in plasma total carotenoids, α- and γ-tocopherol, and vitamin C had no effect on these outcomes. Although the changes in plasma folate and magnesium were proportionally higher with the higher walnut dose, no significant within- or between-group differences were observed for either of these nutrients. The magnitude of change in plasma PLP levels after 6 wk was similar with both doses, i.e., 19.0% with 21 g/d and 16.2% with 42 g/d. However, only the change attained with the higher dose was statistically significant compared to the baseline PLP level for this intervention (P = 0.045).

**Table 5:** Changes in plasma micronutrient status following 6 wk intervention with daily walnut consumption^1^

	Walnut intervention dose				
		
	21 g/d		42 g/d		
			
Micronutrient	% Change from baseline	P (within group)	% Change from baseline	P (within group)	P (between group)
Total carotenoids	-2.9 ± 12.7	0.308	5.2 ± 16.9	0.175	0.110
α-tocopherol	-5.8 ± 22.6	0.25	4.6 ± 17.1	0.229	0.099
γ-tocopherol	-5.9 ± 25.7	0.303	-2.0 ± 29.4	0.762	0.651
α-tocopherol:PUFA	-7.2 ± 22.3	0.157	5.4 ± 26.1	0.356	0.128
γ-tocopherol:PUFA	-7.8 ± 24.0	0.152	0.2 ± 39.4	0.985	0.451
Vitamin C	13.0 ± 67.1	0.386	10.6 ± 51.9	0.363	0.892
Folate	2.3 ± 29.6	0.752	3.6 ± 27.0	0.555	0.877
Vitamin B6 (PLP)	19.0 ± 63.6	0.186	16.2 ± 34.7	0.045	0.859
Magnesium	1.2 ± 8.2	0.517	2.5 ± 6.1	0.086	0.559

^1^Values are means ± SD

**Figure 2:**
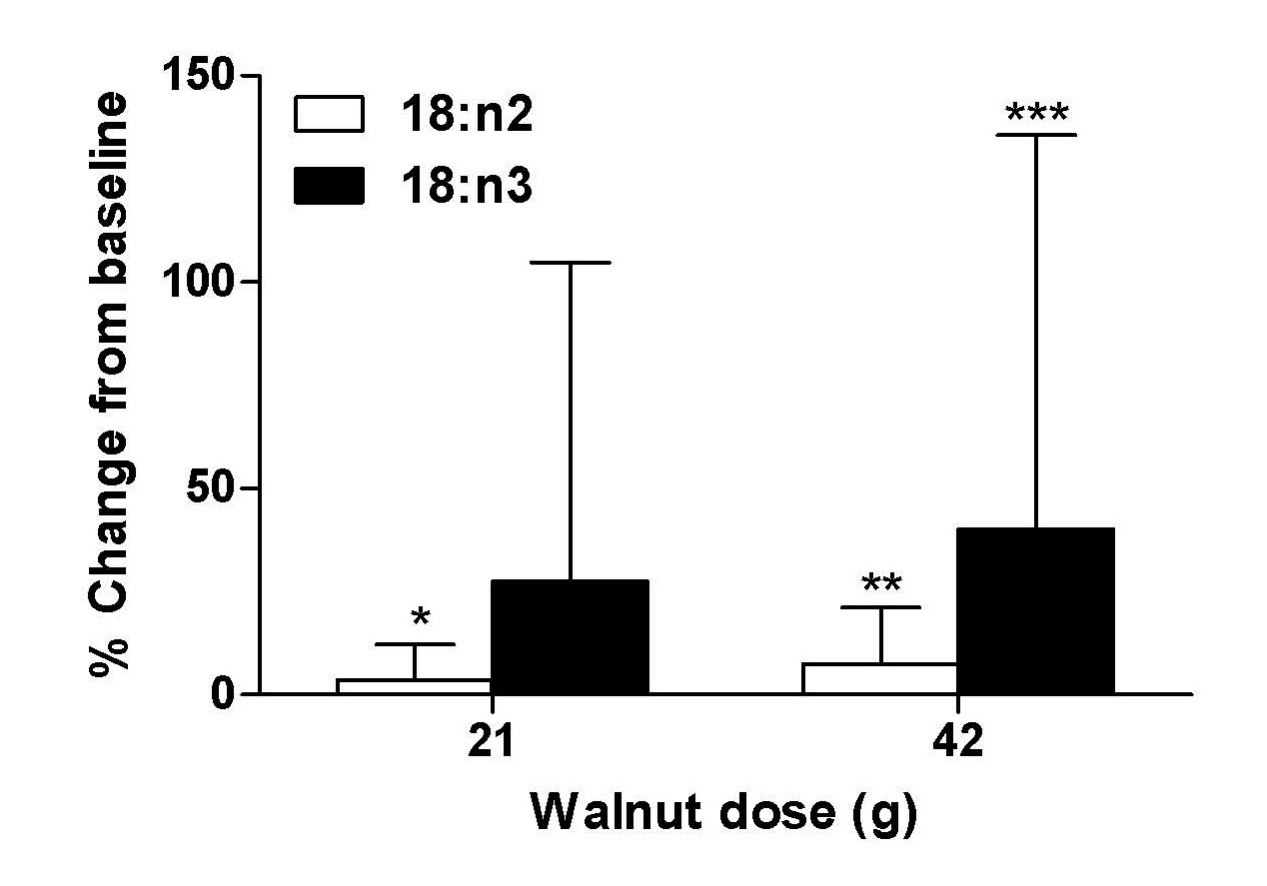
**Changes in RBC linoleic acid and linolenic acid following 6 wk walnut consumption**. Mean baseline RBC 18:n2 was 12.3 ± 1.4 and 11.4 ± 2.0 mol % total fatty acids, and 18:n3 was 0.65 ± 0.64 and 0.52 ± 0.28 mol % total fatty acids for 21 and 42 g/d, respectively. Using paired t-tests, P > 0.05 for between group differences; change from baseline: *P = 0.066, **P = 0.022, ***P = 0.068.

RBC linoleic and linolenic acids were increased after 6 wk with both walnut doses (Fig 2). Compared to baseline levels, the magnitude of change in linoleic acid was significant after 6 wk with 42 g/d. Interestingly, plasma total cholesterol, LDL, and triglyceride levels decreased significantly compared to baseline levels after 6 wk with the 21 g/d dose, but not with the higher dose (Table 6).

**Table 6:** Changes in plasma lipid profile following 6 wk intervention with daily walnut consumption^1^

	Walnut intervention dose				
		
	21 g/d		42 g/d		
			
Lipids	% Change from baseline	P (within group)	% Change from baseline	P (within group)	P (between group)
Total cholesterol	-5.9 ± 7.6	0.002	-1.7 ± 8.6	0.386	0.115
LDL	-6.5 ± 9.5	0.005	-0.5 ± 11.5	0.853	0.097
HDL	-3.7 ± 9.4	0.086	-1.6 ± 9.5	0.449	0.496
Total/HDL	-1.9 ± 7.5	0.264	0.3 ± 7.7	0.846	0.376
Triglycerides	-9.1 ± 18.3	0.034	-0.9 ± 23.5	0.871	0.251

^1^Values are means ± SD

## Discussion

Our study is consistent with the results reported by Davis et al. [11] that chronic walnut consumption has little effect on antioxidant capacity in humans when measured with ORAC, and extends the list of null data to include ORAC-pca, FRAP, and TAP. ORAC-pca measures the inhibitory capacity of small hydrophilic antioxidant molecules alone against free radicals, i.e., absent the contribution of plasma proteins, while FRAP measures the ferric reducing ability of antioxidant compounds present in plasma. The TAP assay assesses the activities of both lipophilic and hydrophilic antioxidants when radicals are formed in hydrophobic cell compartments. The increased linoleic and linolenic acid content confirmed dietary compliance. Despite the observed increase in RBC PUFA associated with walnut consumption, this substrate for lipid peroxidation only minimally affected MDA and antioxidant capacity.

The in vivo antioxidant activity of walnuts in this and other studies may be reflected by the protection of susceptible substrates against free radical attack. Thus, while the shift to more PUFA in the diet and plasma with walnut consumption could be predicted to increase plasma biomarkers of lipid peroxidation, we found no change in MDA. The absence of an increase in MDA may be a result of the concurrent intake and bioavailability of walnut phytochemicals, including phenolic compounds and γ-tocopherol. Anderson et al. [6] demonstrated that walnut polyphenols inhibit AAPH-mediated oxidation of plasma and LDL and spare endogenous LDL α-tocopherol, in vitro. A reduction or null effect of walnut consumption on lipid peroxidation is consistent with the findings of other human intervention studies. For example, Zambon et al. [29] found that 41-56 g/d of walnuts for 6 wk increased γ-tocopherol, but had no effect on LDL α-tocopherol or on the resistance of LDL against Cu^2+^-induced oxidation, when compared with a control diet. Similarly, Ros et al. [30] found an increase in γ-tocopherol with the consumption of 40-65 g/d walnuts for 4 wk, but no change in either the lag time of LDL oxidation, MDA production or on number of oxidatively modified LDL particles when compared with a control diet. Haddad et al. [31] showed a small, but significant increase in γ-tocopherol and decrease in MDA, but no significant change in FRAP or TEAC after 4 wk with 67 g/d of pecans.

Although chronic walnut consumption in this pilot study may not have a readily apparent and marked impact on antioxidant capacity in healthy older adults, its acute postprandial action to increase plasma total thiols is noteworthy. Interestingly, Jenkins et al. [32] observed an increase in plasma total thiols following the consumption of a meal containing almonds in contrast to decreases in this element of antioxidant defenses after meals containing bread, rice, or potatoes. These findings suggest that nuts may reduce postprandial oxidative damage to proteins. Postprandial increases in biomarkers of oxidative damage and decreased antioxidant defenses (including SH groups) have been reported in both diabetic and healthy subjects [33]. This evidence suggests that episodic postprandial increases in oxidative stress may contribute to the pathogenesis and complications of several chronic diseases. Indeed, Ashfaq et al. [34] have reported a direct correlation between thiol biomarkers of oxidative stress and atherosclerosis risk in healthy adults, independent of traditional risk factors and inflammation.

The increase in RBC linoleic and linolenic acids we observed with walnut consumption is consistent with the findings of Rajaram et al. [35] who fed subjects a daily dose of 1.50 oz (42 g)/2400 kcal for 6 wk. However, their study employed a controlled feeding protocol in which all other n-3 fatty acid-rich foods (including fatty fish) were excluded during the walnut intervention. Subjects in our study were not instructed to limit their consumption of these particular foods, so the magnitude of change we expected with the addition of walnuts alone was lower. Despite this attenuated response, our study more accurately reflects the effects of incorporating walnuts into the diet of the general population. In addition, baseline linoleic acid levels in our subjects were similar to the levels achieved by subjects in the Rajaram study [35] following the 6 wk walnut diet, while our baseline linolenic acid levels were more than double their post-intervention levels. The higher baseline nutritional status of our subjects likely also contributed to their smaller change in magnitude. The lack of a significant change in plasma γ-tocopherol following walnut consumption may be due to the continuing use of corn and soybean oils among the subjects. Interestingly, PLP increased with both walnut doses in this study, despite no reported increase in protein consumption from either animal or vegetable sources.

There are several limitations to this pilot study, including its small sample size, the absence of a placebo group, and lack of control over subjects' background diet during the intervention. The sufficiency of nutrient intake and status in our population may have also affected the main study outcomes, as it is difficult to markedly increase plasma nutrient concentrations and measures of antioxidant capacity in subjects who are already well-nourished. Although increases in oxidative stress status and reductions in antioxidant capacity are associated with advancing age, the quality of the subjects' habitual diet may have reduced our ability to detect changes in these outcomes with the addition of walnuts alone. Nonetheless, this study largely confirms earlier observations of the effect of walnuts in human studies and extends our knowledge of their putative role in health maintenance and disease prevention.

## Conclusions

Walnut consumption did not significantly change the plasma antioxidant capacity of healthy, well-nourished older adults in this pilot study. However, improvements in linoleic acid and pyridoxal phosphate were observed with chronic consumption, while total plasma thiols were enhanced acutely. Future studies investigating the antioxidant effects of walnuts in humans are warranted, but should include either a larger sample size or a controlled feeding intervention.

## Abbreviations used

RBC: red blood cell; LDL: low density lipoprotein; PUFA: polyunsaturated fatty acids; ORAC: Oxygen Radical Absorbance Capacity; ORAC-pca: ORAC with protein precipitation by perchloric acid; FRAP: Ferric Reducing Antioxidant Power; TAP: Total Antioxidant Performance; TRAP: Total Radical-trapping Antioxidant Parameter; TEAC: Trolox Equivalent Antioxidant Capacity; CVD: cardiovascular disease; BMI: body mass index; EKG: electrocardiogram; DV: daily value; MDA: malondialdehyde; PLP: pyridoxal phosphate; GPX: glutathione peroxidase; HDL: high density lipoprotein; EDTA: ethylenediaminetetraacetic acid; BHT: butylated hydroxytoluene; RBC: red blood cells; AAPH: 2,2-azobis(2-amidopropane) hydrochloride; TE: Trolox equivalents; MeO-AMVN: 2,2'-azobis(4-methoxy-2,4-dimethylvaleronitrile); BODIPY: 4,4-difluoro-4-bora-3a,4a-diaza-s-indacene; TBA: thiobarbituric acid; HPLC: high pressure liquid chromatography; GAE: gallic acid equivalents; SD: standard deviation; FDA: Food and Drug Administration; α: alpha; β: beta; γ: gamma

## Competing interests

The authors declare that they have no competing interests.

## Authors' contributions

DLM and JBB conceived of the study design and obtained funding; C-YOC performed biochemical analyses for antioxidant activity markers; K-JY performed TAP analyses; NM supervised the fatty acid analyses; DLM conducted the study, performed the statistical analyses, drafted the manuscript, and had primary responsibility for its final content. AHL provided critical input during manuscript preparation. All authors read and approved the final manuscript.
